# Clinical evaluation of a nutraceutical diet as an adjuvant to pharmacological treatment in dogs affected by Keratoconjunctivitis sicca

**DOI:** 10.1186/s12917-016-0841-2

**Published:** 2016-09-22

**Authors:** Simona Destefanis, Daniela Giretto, Maria Cristina Muscolo, Alessandro Di Cerbo, Gianandrea Guidetti, Sergio Canello, Angela Giovazzino, Sara Centenaro, Giuseppe Terrazzano

**Affiliations:** 1 Clinica Veterinaria Porta Venezia, via Lambro 12, 20121 Milan, Italy; 2 Clinica Veterinaria Cartesio, viale Olanda 3B, Melzo, 20066 Milan, Italy; 3 Ambulatorio Veterinario Canonica, via Canonica 36, 20154 Milan, Italy; 4 School of Specialization in Clinical Biochemistry, “G. d’Annunzio” University, Chieti, Italy; 5 Research and Development Department, SANYpet S.p.a., Bagnoli di Sopra, Padua, Italy; 6 Department of Science, University of Basilicata, Via Sauro, 85, 85100 Potenza, Italy; 7 Department of Translational Medical Sciences, University of Naples Federico II, Via Pansini, 5, 80131 Naples, Italy

**Keywords:** Antioxidant and anti-inflammatory diet, Immune-mediated ocular disease, Keratoconjunctivitis sicca, Nutraceutical diet

## Abstract

**Background:**

Canine keratoconjunctivitis sicca (cKCS) is an inflammatory eye condition related to a deficiency in the tear aqueous fraction. Etiopathogenesis of such disease is substantially multifactorial, combining the individual genetic background with environmental factors that contribute to the process of immunological tolerance disruption and, as a consequence, to the emergence of autoimmunity disease. In this occurrence, it is of relevance the role of the physiological immune-dysregulation that results in immune-mediated processes at the basis of cKCS. Current therapies for this ocular disease rely on immunosuppressive treatments. Clinical response to treatment frequently varies from poor to good, depending on the clinical-pathological status of eyes at diagnosis and on individual response to therapy. In the light of the variability of clinical response to therapies, we evaluated the use of an anti-inflammatory/antioxidant nutraceutical diet with potential immune-modulating activity as a therapeutical adjuvant in cKCS pharmacological treatment. Such combination was administered to a cohort of dogs affected by cKCS in which the only immunosuppressive treatment resulted poorly responsive or ineffective in controlling the ocular symptoms.

**Results:**

Fifty dogs of different breeds affected by immune-mediated cKSC were equally distributed and randomly assigned to receive either a standard diet (control, *n* = 25) or the nutraceutical diet (treatment group, *n* = 25) both combined with standard immunosuppressive therapy over a 60 days period. An overall significant improvement of all clinical parameters (tear production, conjunctival inflammation, corneal keratinization, corneal pigment density and mucus discharge) and the lack of food-related adverse reactions were observed in the treatment group (*p* < 0.0001).

**Conclusions:**

Our results showed that the association of traditional immune-suppressive therapy with the antioxidant/anti-inflammatory properties of the nutraceutical diet resulted in a significant amelioration of clinical signs and symptoms in cKSC. The beneficial effects, likely due to the presence of supplemented nutraceuticals in the diet, appeared to specifically reduce the immune-mediated ocular symptoms in those cKCS-affected dogs that were poorly responsive or unresponsive to classical immunosuppressive drugs. These data suggest that metabolic changes could affect the immune response orchestration in a model of immune-mediated ocular disease, as represented by cKSC.

## Background

Keratoconjunctivitis sicca, also defined as “dry eye disease” or Sjögren’s syndrome in human [1], is a tear film disorder which causes inter-palpebral ocular surface damage and is associated with ocular discomfort [2, 3] both in humans and dogs [4, 5]. Canine keratoconjunctivitis sicca (cKCS) is an inflammatory eye condition which affects both cornea and conjunctiva and that is related to a deficiency in tear aqueous fraction [6]. The prevalence of such disease is estimated in about 4 % when considering Schirmer test I (STT) values < 10 mm/min [7] reaching the 64 % in male crossbred dogs between six to nine years of age [8]. Moreover, it is often an under-recognized and/or a sub-clinical condition [9] which, in some breeds, is preceded by an immune-mediated destruction of lachrymal glands [10, 11].

In this regard, the immune-mediated mechanisms of cKCS or of human, like the Sjögren’s syndrome [1] induction are not clearly defined. Etiopathogenesis of such disease is substantially multifactorial, combining the individual genetic background with environmental factors that contribute to the process of immunological tolerance disruption and, as consequence, to the autoimmunity processes [12–14]. It is of relevance the role of the physiologic immune-dysregulation that results in the autoimmune process of cKCS and Sjögren’s syndrome [12–15]. Notably, the T and B cell infiltration, the recruitment of dendritic cells, the up regulation of those molecules fostering the antigen presentation as well as the increased secretion of pro-inflammatory cytokines, such as interferon (INF)-γ [16], in ocular tissues have been demonstrated to contribute to the inflammatory alterations of the lachrymal gland [17–19]. This process usually results in mucopurulent-like eye discharge, conjunctival hyperemia, keratitis, corneal pigmentation, neovascularization and blepharospasm in cKCS [20, 21].

Current therapies for this ocular disease rely on immune-suppressive treatments, represented by Cyclosporine A [22], glucorticoid [21], tacrolimus [23] and artificial tears in order to recover an adequate eye’s lubrication [24]. Nevertheless, recognized complementary or alternative therapeutical approaches are represented by the cholinergic agents (pilocarpine) [25] and the surgical treatments (punctal occlusion, tarsorrhaphy, conjunctival flaps, contact lenses, superficial keratectomy, as well as parotid duct transposition) [26]. Clinical response to treatment frequently varies from poor to good, depending on the clinical-pathological status of eyes at diagnosis and on individual response to therapy [13]. Among other causes of cKCS traumas [27], congenital causes [28], distemper [29], radiation therapy [30, 31], neurological deficit [32], diabetes mellitus [33] and uncorrected prolapse of the nictitans gland [34] are of note. Intriguingly, majority of these aspects could correlate and contribute to both the determinism and exacerbation of inflammatory condition in ocular tissue.

In the light of the variability of clinical response to classical therapies, it could be useful the use of therapeutical adjuvants in cKCS management to improve the response to pharmacological treatment. Thus, we evaluated a combined therapeutical approach based on the classical drug administration and the use of an anti-inflammatory/anti-oxidant diet with potential immune-modulating activity. Such combination was administered to a cohort of cKCS dogs in which the only immune-suppressive treatment resulted poorly responsive or ineffective to control the ocular symptoms.

The nutraceutical diet used in this clinical evaluation consisted in a commercial mixed formula based on fish proteins, rice carbohydrates (whose carbohydrates percentage ranges from 75 up to 80, starch 65 to 70 % with a beta-glucans quote of less than 0.1 %), *Cucumis melo*, *Ascophyllum nodosum*, Astaxanthin (from *Hematococcus pluvialis*), *Aloe vera*, *Carica papaya*, *Punica granatum*, *Camellia sinensis*, *Polygonum cuspidatum*, *Curcuma longa*, *Piper nigrum*, zinc and a Omega3/6 ratio of 1:0.8), which already provided significant immunomodulating results, decreasing type 1 helper T lymphocyte (Th1) cells and increasing T regulatory (Treg) cells, in dogs affected by *Leishmania infantum* [35].

*Cucumis melo* (melon) shares some anti-oxidant and anti-inflammatory properties that involve the superoxide/peroxynitrite clearance and the modulation of macrophagal interleukin-10 production [36], while the immune-modulating activity is exerted by the induction of type 1 helper T lymphocyte (Th1) polarization [37].

The *Ascophyllum nodosum* activity is related to the presence of a sulfated-polysaccharide, ascophyllan, able to induce nitric oxide, tumor necrosis factor (TNF)-α and granulocyte colony-stimulating factor (GM-CSF) secretion in macrophages [38]. Astaxanthin, an orange-pinkish carotenoid, is known to act on polyunsaturated fatty acids oxidation [39], inflammatory responses modulation, and to promote eye’s health in humans and animals [40]. This carotenoid induces lymphoblastogenesis and lymphocyte cytotoxicity in mice [41] as well as T-cell and B lymphocyte proliferation and natural killer cytotoxicity in humans [42]. Reduced production of Interleukin (IL)-1β, IL-6, TNF-α and IL-10 has been observed in vitro after the addition of *Aloe vera* (aloe) extracts to the culture of corneal cells [43]. The anti-inflammatory effect of *Carica papaya* (papaya) is related to an increase of regulatory T cells and a reduction of IFN-γ^+^ CD4^+^ T cells [44]. Reduction of IL-2 and IL-4 and enhancement of IL-12, interferon (IFN)-γ and TNF-α have been observed in blood mononuclear cells [45]. The seed oil and juice of *Punica granatum* (pomegranate) contains some flavonoids and anthocyanidins (delphinidin, cyaniding and pelargonidin) with an antioxidant activity greater than green tea extract [46, 47]. Its antioxidant action is related to free radical scavenging by anthocyanidins [46] and to metal ions chelation [48]. A protective effects of *Punica granatum* on cardiovascular system has been correlated to angiotensin converting enzyme inhibition, blood pressure decrease [49] and endothelial nitric oxide syntase production [50]. *Punica granatum* also has been shown to inhibit cyclooxygenase, lipooxygenase [51] and IL-1β, modulate matrix metallo-proteinases in osteoarthritis, prevent collagen degradation [52], inhibit the p38-mitogen-activated protein kinase pathway and nuclear factor kappa (NF-kB) light-chain-enhancer in B cells [53, 54], and decrease malondialdehyde, TNF-*α*, IL-1β and IL-6 [55, 56].

The antioxidant effects of *Camellia sinensis* (green tea) are exerted through radicals scavenging and lipid-peroxidation inhibition [57] by flavonoids (catechin, epicatechin, epigallocatechin and gallate esters) [58]. In this context, epigallocatechin-3-gallate is known to inhibit UVB-mediated erythema, hydrogen peroxide production, leukocyte infiltration [59], matrix metallo-proteinases [60, 61], neutrophil chemotaxis [62], degradation of cartilage [63], TNF-α expression [64], neutrophil-mediated angiogenesis [62] and reduce the cyclooxygenase-2 and neutral endopeptidase activity [65]. *Polygonum cuspidatum* (japanese knotweed), a natural source of resveratrol, is endowed with anti-inflammatory and antioxidant activities [66, 67]. Resveratrol has been shown to directly act on TANK-binding kinase 1, an integral component in chronic inflammatory diseases [68], and on arteries by activating the nitric oxide/soluble guanylyl cyclase pathway [69]. Its anti-inflammatory effect is supposed to be regulated by estrogen receptor-α [70]. Moreover, certain resveratrol dimers (parthenocissin A, quadrangularin A and pallidol) exert free radical quenching and, selectively, single oxygen scavenging activity [71]. *Curcuma longa* (curcuma) induces powerful free radicals scavenging effect and anti-inflammatory activity [72, 73]. Curcumin, one of the constituents of such plant, reduces leukocyte adhesion and superoxide production, stimulates spontaneous apoptosis and inhibits IL-8 [74].

Moreover, a down regulation of Th1 cytokine response and of macrophagal nitric oxide production has also been observed [75]. The anti-inflammatory effect of curcumin involves the inhibition of NF-kB in activated B cells and the down-regulation of TNF-α and IL-6 [73] as well as the up-regulation of nuclear factor erythroid 2 activity [76], whose downstream proteins are involved in the protection mechanisms against oxidative stress [77]. *Piper nigrum* (pepper) commonly used in the treatment of flu, cold, rheumatism, pain, muscular aches, chills, exhaustion, fevers, is used as a useful nerve tonic also able to increase blood circulation and saliva production as well as to stimulate appetite and peristalsis [78]. It is also known to enhance the effectiveness and bioavailability of curcumin [79] by acting on membrane lipid dynamics in reason of the apolar nature of piperine, the main bioactive compound of *Piper nigrum*. Piperine has been shown to promote conformational changes of intestine enzymes [80] and significantly inhibit the expression of major histocompatibility complex class II, CD40 and CD86 in bone-marrow-derived dendritic cells as well as the production of TNF-α and IL-12 by the same cells [81]. In addition, piperine was proven to attenuate inflammatory processes by partially acting on pituitary adrenal axis [82], reduce high-fat diet-induced oxidative stress [83, 84] and enhance pancreatic activity [85]. The deficiency of zinc affects both innate and adaptive immunity [86]. This element is crucial for the balance between the different T-cell subsets and its deficiency was shown to decrease the production of Th1 cytokines (IFN-γ, IL-2 and TNF-α), whereas the Th2 response (IL-4, IL-6 and IL-10) is affected in a lesser extent [87]. While acute zinc deficiency seems to correlate with the decrease in innate and adaptive immunity, its chronic deficiency is known to increase pro-inflammatory cytokines (IL-1β, IL-6 and TNF-α) production influencing the outcome of several inflammatory diseases [88].

An optimal balance of the omega Omega 3/6 fatty acids ratio represents a fundamental requirement for tissue homeostasis recovering during inflammatory responses. The polyunsaturated fatty acids, usually found in fish oil (i.e., eicosapentaenoic acid and docosahexaenoic acids), are known to decrease proinflammatory cytokine production and to inhibit natural killer cell activity [89]. The gamma-linolenic acid has been demonstrated to exert an anti-inflammatory activity by suppressing IL-1β and TNF-α secretion by monocytes [90]. Additionally, eicosapentaenoic supplementation might foster the anti-inflammatory activity of gamma-linolenic acid by decreasing the synthesis of arachidonic acid and prostaglandin E2 [91].

Here, we evaluated the use of a commercially available nutraceutical diet as a therapeutical adjuvant in cKCS-affected dogs that were unresponsive to standard pharmacological therapies.

## Methods

### Experimental design, dogs and diets

This evaluation was designed as a randomized, placebo-controlled clinical one. Fifty client-owned dogs (19 females and 31 males) aged 6.5 ± 0.7 years [mean ± Standard Error of Mean] of different breeds (one poodle, two dachshund long hair, four dachshund smooth coat, four west highland white terrier, two yorkshire terrier, four maltese, one bulldog, two chinese crested dog, two chinese pug, eight shih tzu, four german shepherd, 10 mixed breed, two chow chow, two cocker, two english setter) were enrolled in this evaluation. All dogs were previously evaluated by an Italian Animal Health Foundation certified panelist (Dr D. Giretto) to confirm the diagnosis of immune-mediated KCS. Inclusion criteria were the presence of blepharospasm, conjunctival inflammation, corneal keratinization, corneal pigmentation density, neovascularization, mucus discharge and a STT value < 10 mm/min. Exclusion criteria were the presence of correlated systemic diseases, neurological disease, traumatic and toxic keratoconjunctivitis, in order to better evaluate the clinical response to the immune-mediated cKCS, or general symptoms of intolerance/allergy to ingredients of the nutraceutical diet tested in this clinical evaluation. Moreover subjects affected by neurological cKCS were excluded.

Dogs were randomly and equally divided into two groups: 25 dogs fed a standard diet (SD group), as control group, and 25 fed an antioxidant/anti-inflammatory nutraceutical diet (ND group), as experimental group. Male and female dogs were equally represented in both groups. Regardless the type of diet, all dogs were treated over a 60 days period as follows: [0,03 % Tacrolimus collyrium diluted into a benzalkonium chloride and methyl cellulose solution (Lacrimart, Fedel Farma S.r.l., Chieti, Italy) BID and 0,2 % Hyalistil eye drops (artificial tears, S.I.F.I. S.p.A. Aci S. Antonio, Catania, Italy) five times a day] ([http://eng.forza10.com/immuno-active-755-2.html]).

The recommendations of the ARRIVE guidelines in animal research were consulted and considered [92].

In Table 1, we reported the background data of the dogs belonging to both groups along with their scores before starting the evaluation.

**Table 1:** Background data of enrolled dogs

Group	Mean age (years ± SEM)	Mean weight (Kg ± SEM)	STT value (mm ± SEM)	Corneal pigment density score (0-3 ± SEM)	Conjunctival inflammation score (0-3 ± SEM)	Mucus discharge score (0-3 ± SEM)	Corneal keratinization score (0-2 ± SEM)
Control	6.03 ± 0.15	13.04 ± 1.12	4.3 ± 0.5	1.0 ± 0.1	2.1 ± 0.1	1.7 ± 0.1	1.5 ± 0.1
Treatment	6.1 ± 0.17	12.01 ± 1.17	4.7 ± 0.4	0.9 ± 0.1	2.1 ± 0.1	1.8 ± 0.1	1.5 ± 0.1

Both diets completely fulfil the recommendations for proteins, carbohydrates and fats in order to obtain a complete food for a daily ration in dog, as reported in Nutritional Guidelines for complete and complementary pet food for cats and dogs by The European Pet Food Industry Federation. Foods were in the form of kibbles industrially produced with extrusion technique. ND and SD foods reported similar analytical composition in nutrients (24 % of crude protein, 12 % of crude oils and fats, 3.7 %, of crude fiber 5 % of crude ash, 9 % of moisture). Both diets had analogue recipes and included the same macro and micro nutrients including vitamins, trace elements and minerals. The two foods differed mainly from the presence of botanicals in ND food. ND food was composed by two mixed components: kibbles, included in the ideal percentage of 93-94 % in weight, and cold-pressed tablets at the 6-7 % in weight of complete food (European patent n. EP 2526781). Tablets were composed by 60-80 % of protein hydrolyzed (fish and vegetable ones), 20-40 % of minerals used as glidants and were added by therapeutical substances (*Ascophyllum nodosum, Cucumis melo, Carica papaya, Aloe vera, Astaxanthin from Haematococcus pluvialis, Curcuma longa, Camellia sinensis, Punica granatum, Piper nigrum, Poligonum spp, Echinacea purpurea, Grifola frondosa, Glycine max*, Omega 3 and Omega 6 un-saturated fatty acids from fish, as 1.60 % and 1.25 % of oil respectively).

The pet food used in SD group did not contain the above-mentioned active substances.

ND and SD dietary administration were administrated following a daily table recommendation (Table 2) and carefully adjusted during the trial to provide similar caloric animal food intake and to satisfy the nutritional requirement of adult dogs. In order to avoid any deficiency, the energy value of both complete food was calculated using the expression suggested by Nutritional Guidelines for Complete and Complementary Pet Food for Cats and Dogs and Nutrient requirements of dogs and cats, National research council of the National academies, (% crude protein x 3.5 + % crude fat x 8.5 + % NFE (Nitrogen-free extract) × 3.5). The correct dosage was calculated using another expression 110 kcal ME*kg bw^0.75^ (Nutritional Guidelines for Complete and Complementary Pet Food for Cats and Dogs and Nutrient requirements of dogs and cats, National research council of the National accademies). The constant 110 is referred to the energy requested by a dog with normal physical activity. At the enrollment, each animal was weighed and the suggested daily ratio calculated. The Veterinarians clearly informed the owners about the correct dosage to be provided. Moreover the average of daily administered botanicals was calculated considering the ratio given to the dogs, related to the amount declared by the manufacturer. Table 3 highlights the average amount, in terms of mg/kg, of botanicals estimated according to the mean weight.

**Table 2:** Daily table recommendation for diet

Weight (Kg)	Diet amount per day (g)
1 – 10	30 – 180
11 – 20	190 – 300
21 – 35	310 – 455
36 – 50	465 – 595

**Table 3:** Average substances administer to dog depending on body weight (considering medium body weight)

Nutraceutical substances	Amount per kg of complete food		Dog weight 10 kg	11 kg	12 kg	13 kg
Ascophyllum nodosum	40000	mg/kg	7200	7600	8200	8600
Cucumis melo	300	mg/kg	54	57	61,5	64,5
Carica papaya	135	mg/kg	24,3	25,65	27,675	29,025
Aloe vera	135	mg/kg	24,3	25,65	27,675	29,025
Haematococcus pluvialis (astaxanthin)	49	mg/kg	8,82	9,31	10,045	10,535
Resveratrol (Poligonum Cuspidatum)	7	mg/kg	1,26	1,33	1,435	1,505
Zinc sulphate monohydrate	137	mg/kg	24,66	26,03	28,085	29,455
Curcuma longa	102	mg/kg	18,36	19,38	20,91	21,93
Camellia sinensis	70	mg/kg	12,6	13,3	14,35	15,05
Punica granatum	70	mg/kg	12,6	13,3	14,35	15,05
Piper nigrum	30	mg/kg	5,4	5,7	6,15	6,45

### Ophthalmologic examination

Each dog was evaluated on day 0,15, 30, and 60 of the evaluation by an independent observer (SD, DG, CM). Each dog underwent a complete ophthalmological examination by three board-certified veterinary ophthalmologists (Dr. M.C. Muscolo and Dr. S. De Stefanis are board-certified by the D’Ophtalmologie ENV Alfor; Dr. D. Giretto is board-certified by Certificat d’Etudes Superieur en Ophtalmologie ENV Toulouse and is an Italian Animal Health Foundation board member).

Ophthalmic examinations included, slit-lamp biomicroscopy (Kowa Optimed Inc SL-14 Slit Lamp, Kowa Optimed, Europe Ltd, Berkshire, UK), funduscopic examination (Heine Omega 180 Binocular Indirect Ophthalmoscope, HEINE Optotechnik, Herrsching, Germany), applanation tonometry (Tono-Pen^®^ Vet, Reichert Technologies, Depew, NY, USA) preceded by an ocular application of oxybuprocaine hydrochloride 0.4 % (Novesina Novartis Farma S.p.A, Origgio (VA), Italy) in order to reduce the nuisanceand fluorescein dye staining (fluorescein 0.5 % collyre unidose TVM, Laboratoires TVM, Lempdes, France) along with 0.9 % physiologic rinsing solution (Eurospital S.p.A., Trieste, Italy).

Both eyes of each dog were photographed at each visit in the afternoon (3–6 pm) and clinical signs, such as corneal pigment density and corneal keratinization, were graded according to the scores proposed by Hendrix et al. [93], whereas conjunctival inflammation and mucus discharge were graded according to the scores proposed by Moore et al. [94].

- corneal pigment density (0-3): 0 = no pigment, 1 = iris easily visualized through the pigment, 2 = iris partially visualized through the pigment, 3 = iris not visible through the pigment);
- conjunctival inflammation (0-3): 0 = normal conjunctiva; 1 = mild hyperemia without chemosis; 2 = moderate hyperemia with mild chemosis; 3 = intense hyperemia with moderate to severe chemosis;
- mucus discharge (0 – 3): 0 = no visible mucus or clear mucus thread; 1 = scattered non-adherent mucopurulent strands; 2 = moderate adherent mucopurulent strands covering up to 25 % of the cornea; and 3 = diffuse extensive adherent mucopurulent discharge covering 25 % to 50 % of the cornea;
- corneal keratinization (0-2): 0 = none, 1 = mild opacity, 2 = moderate opacity.

Enrolled dogs were treated by their owners at home by applying the pharmacological treatment as previously described and the diet administration approximately every 12 h.

### Schirmer tear test

Schirmer tear test-1 (STT-1) is a routine examination which is performed by placing a standard test strip (Schirmer-Plus^®^, Gecis Ecoparc, Domaine de Villemorant, France) within the ventral conjunctival sac of each dog for 60 s. Tear production is then recorded in mm/min for each eye. STT-1 was performed on 100 eyes of dogs of several breeds.

### Statistical analysis

Data were analyzed using GraphPad Prism 6 software (GraphPad Software, Inc., La Jolla, CA, USA). All data are presented as the means ± standard error of the mean and were first checked for normality using the D’Agostino-Pearson normality test. Differences in Schirmer test between the two treatments at the end of treatment versus baseline for each eye were blindly analyzed by ADC using a two-way analysis of variance (ANOVA) followed by Sidak’s multiple comparisons test. Conjunctival inflammation, corneal keratinization, corneal pigmentation density and mucus discharge score between the two treatments at the end of treatment versus baseline for each eye were analyzed using a paired *t*-test. Veterinary ophthalmologists were not involved in the statistical analysis of the data.

## Results and Discussion

### Clinical evaluation of eyes in ND and SD group

Fifty dogs were enrolled in the trial: 25 dogs received the pharmacological treatment and a standard diet (SD Group), while 25 dogs received the pharmacological treatment plus an antioxidant/anti-inflammatory nutraceutical diet (ND Group).

An overall amount of 100 eyes was considered according to literature suggestions [95–97]. All dogs completed the 60-day evaluation period.

**Fig. 1:**
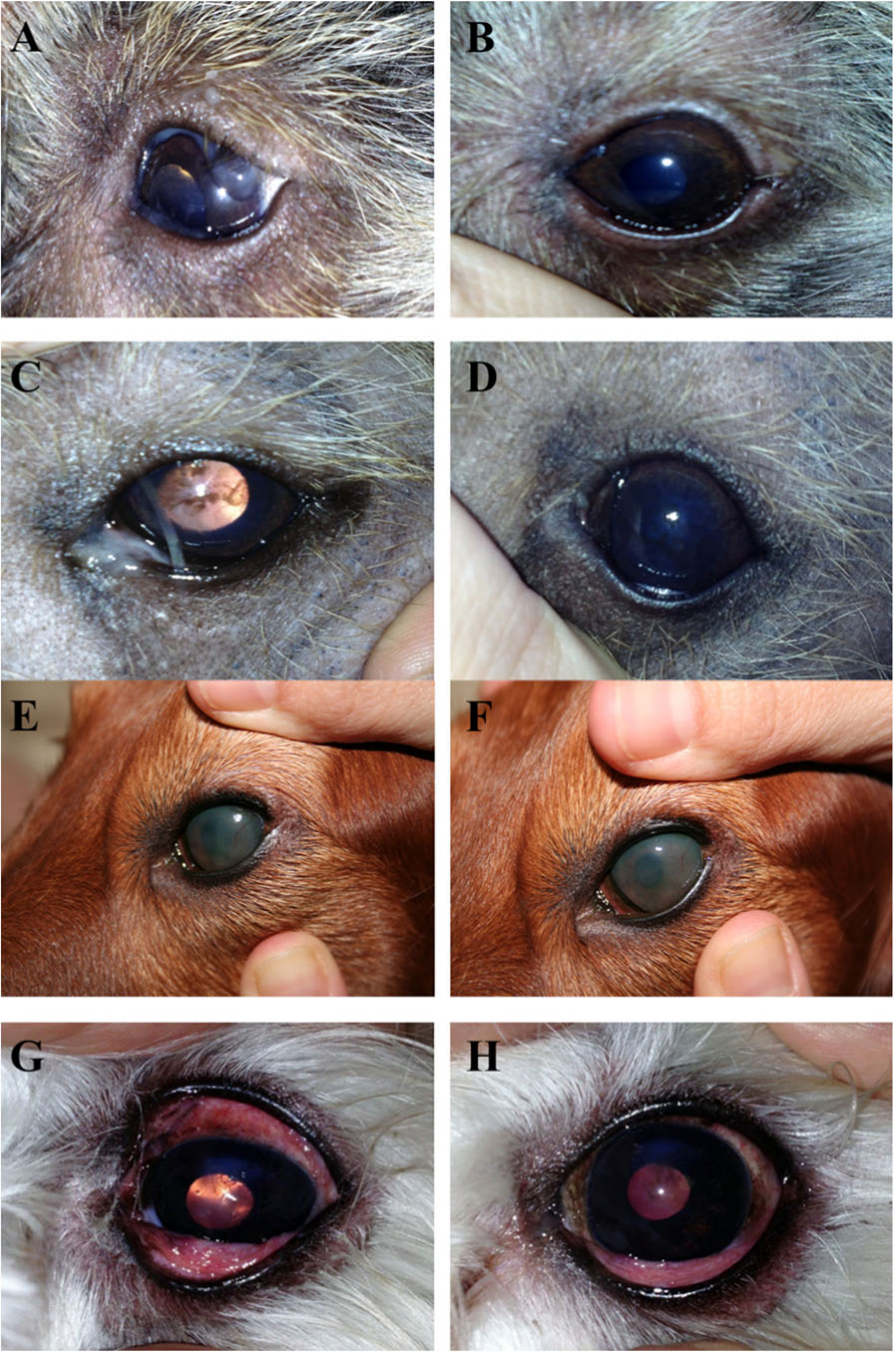
Eyes improvement after 60-days treatment with 0,03 % Tacrolimus collyrium BID and 0,2 % Hyalistil eyewash plus the nutraceutical diet in ND group and with with 0,03 % Tacrolimus collyrium BID and 0,2 % Hyalistil eyewash plus the standard diet in SD group. **a**–**c** eye before treatment plus nutraceutical diet (time = 0), **b**–**d** eye at the end of treatment plus nutraceutical diet (time = 60). **e**–**g** eye before treatment plus standard diet (time = 0), **f**–**h** eye at the end of treatment plus standard diet (time = 60)

The overall improvement of eye’s condition in two representative dogs of ND group at the day 0 of the trial (Fig. 1a, c) and at the end of the 60-days evaluation (Fig. 1b, d) is shown. In particular, our results highlight the clinical amelioration occurred in ND group (Fig. 1b, d) in terms of blepharospasm, ocular hyperemia, periocular swelling and ocular discharge that is strongly dependent on nutraceuticals administration since no effects were evident in SD group (Fig. 1e, h). In this regard, the comparative evaluation between the day 0 (Fig. 1e, g) and the end of 60-days (Fig. 1f, h) in two representative dogs of SD group showed none significant clinical amelioration. Indeed, blepharospasm, ocular hyperemia, periocular swelling and ocular discharge were still evident or, at least, poorly improved.

These results strongly pointed to a specific effect of nutraceuticals in inducing anti-inflammatory and immune-modulating outcomes in eyes of dogs belonging to ND group. Notably, the standard pharmacological treatment appeared to be substantially ineffective since no amelioration has been observed in dogs belonging to SD group. Therefore, the effect of nutraceuticals could be considered as highly fostering the clinical improvement during the pharmacological treatment in cKSC.

### The eye’s scores amelioration in cKSC dogs treated with ND

**Fig. 2:**
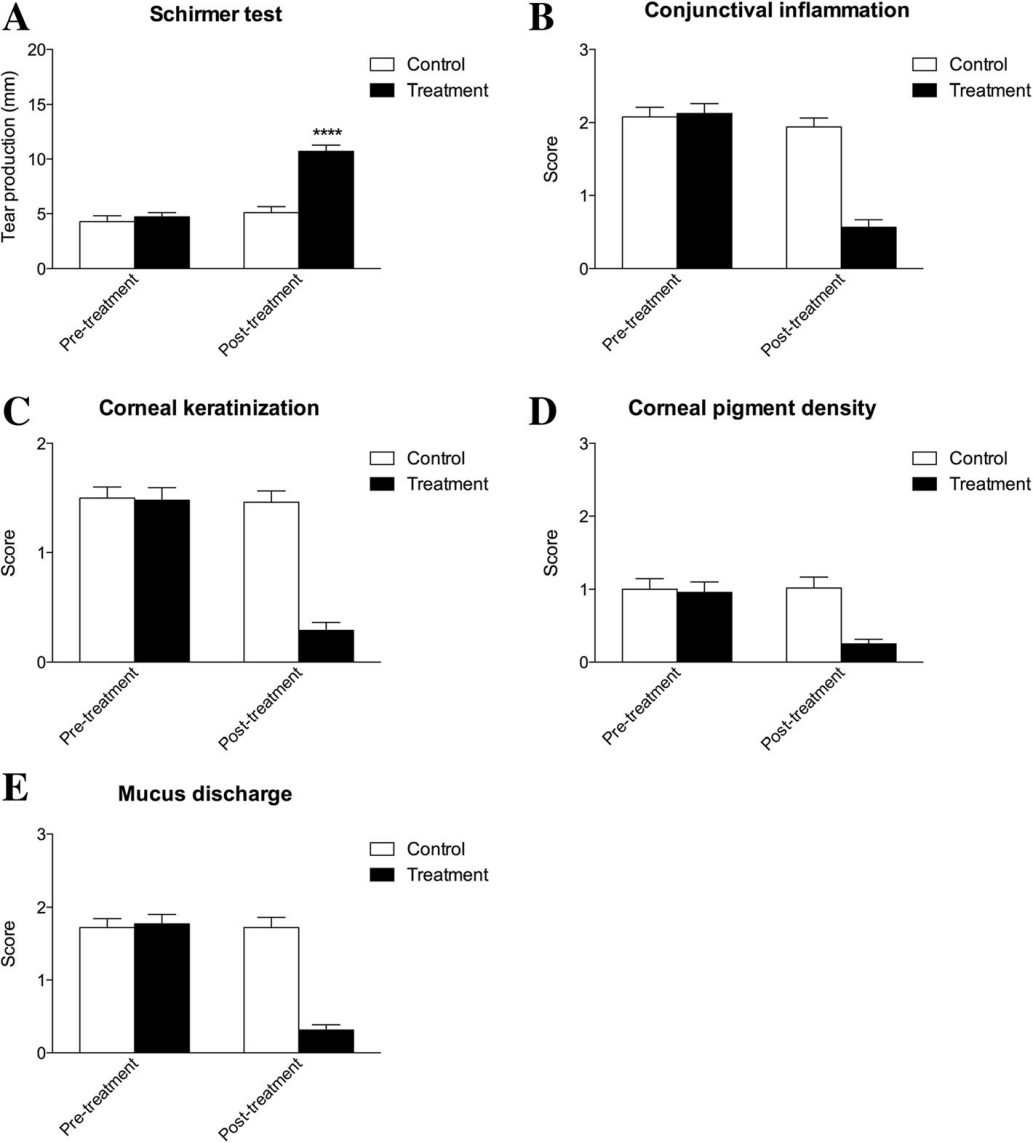
**a**-**b** Mean conjunctival inflammation scores before and after 60 days treatment for ND and SD group, a significant decrease (****P < 0.0001) was observed in ND group at the end of the treatment; **c**–**d** mean corneal keratinization scores before and after 60 days treatment for ND and SD group, a significant decrease (****P < 0.0001) was observed in ND group at the end of the treatment; **e**–**f** mean corneal pigment density scores before and after 60 days treatment for ND and SD group, a significant decrease (****P < 0.0001) was observed in ND group at the end of the treatment; **g**–**h** mean mucus discharge scores before and after 60 days treatment for ND and SD group, a significant decrease (****P < 0.0001) was observed in ND group at the end of the treatment; **i**–**l** mean tear production (STT) in mm/min before and after 60 days treatment for ND and SD group, STT values resulted significantly increased (****P < 0.0001) in ND group at the end of treatment

Figure 2 shows the eye’s score intensity trend of each symptom of dogs belonging to SD and ND group.

Dogs conjunctival inflammation score significantly decreased from a baseline of 2.1 ± 0.1 to 0.6 ± 0.1 in the ND group, while no significant variation (from a score of 2.1 ± 0.1 to 1.9 ± 0.1) appeared in SD group (Fig. 2a–b).

In addition, corneal keratinization score resulted significantly decreased in ND group (from 1.5 ± 0.1 to 0.2 ± 0.1) and not in SD group (from 1.5 ± 0.1 to 1.4 ± 0.1) (Fig. 2c–d). Finally, corneal pigment density and mucus discharge resulted significantly decreased only in ND group, while no effects were evident in SD group. More in details, corneal pigment density scores decreased from a baseline value of 0.9 ± 0.1 to 0.2 ± 0.1 whereas mucus discharge scores decreased from 1.8 ± 0.1 to 0.3 ± 0.1 (Fig. 2e–h).

These results clearly suggest the role for ND in inducing the amelioration of eye’s score testing in cKSC and that this occurrence appears independent on pharmacological treatment since drugs alone appeared ineffective, as evident in SD group.

As to STT-1 values, a significant increase was observed from a baseline value from 4.7 ± 0.4 mm to 10.7 ± 0.6 mm after the 60-days of treatment only in the dogs of ND group, while no significant improvement (STT-1 values from 4.3 ± 0.5 mm to 5.1 ± 0.5 mm) was evident in the dogs of SD Group at the end of the trial (Fig. 2i–l).

These results evidenced the effectiveness of ND in increase the tear film in our cohort of sick dogs. It is reasonable that the anti-inflammatory effects of nutraceuticals could contribute to restore the physiological eye’s tear production in cKSC.

### The relapse/regression of cKSC symptoms in dependence of ND administration

**Fig. 3:**
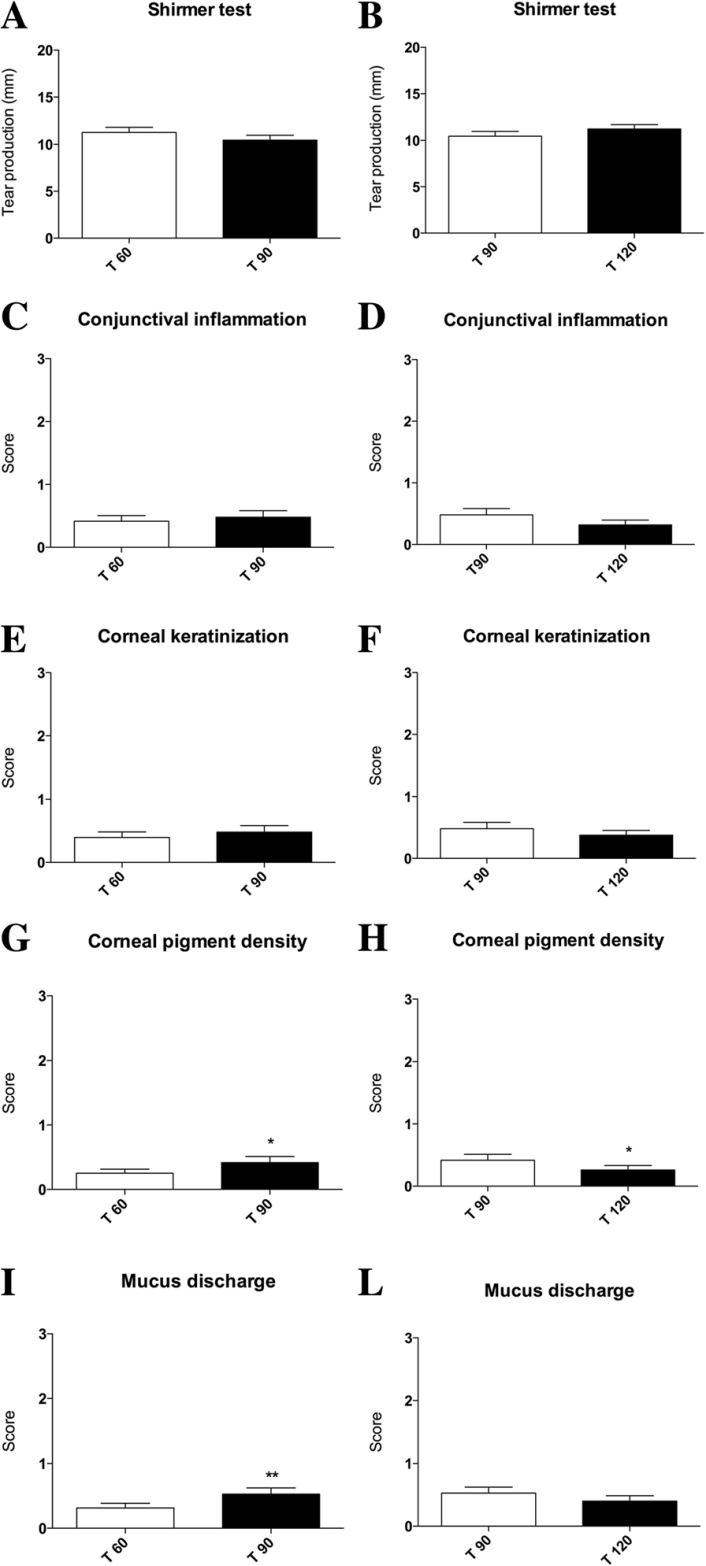
Graphical schematization of clinical symptoms score trends after 30 days since treatment suspension and after 30 days since treatment resumption. **a** Mean conjunctival inflammation scores before (T 60) and after 30 days nutraceutical diet suspension (T 90) for ND group and (**b**) after 30 days since nutraceutical diet resumption (T 120); **c** mean corneal keratinization scores before (T 60) and after 30 days nutraceutical diet suspension (T 90) for ND group and (**d**) after 30 days since nutraceutical diet resumption (T 120); **e** mean corneal pigment density scores before (T 60) and after 30 days nutraceutical diet suspension (T 90) for ND group and (**f**) after 30 days since nutraceutical diet resumption (T 120), scores resulted significantly increased (*P < 0.05); **g** mean mucus discharge scores before (T 60) and after 30 days nutraceutical diet suspension (T 90) for ND group and (**h**) after 30 days since nutraceutical diet resumption (T 120), scores resulted significantly increased (**P < 0.01); **i** mean tear production (STT) in mm/min before (T 60) and after 30 days nutraceutical diet suspension (T 90) for ND group and (**l**) after 30 days since nutraceutical diet resumption (T 120)

After the 60 days of evaluation, dogs belonging to ND group interrupted the diet supplementation for 30 days, while continuing the pharmacological treatment. It is worth noting that a rapid and intensive relapse of symptoms was observed after 15 days since ND suspension. All dogs were newly supplemented with the ND while continuing the pharmacological therapy for another 30 days. Intriguingly, an overall regression of symptoms was again observed after the reintroduction of ND (Fig. 3).

This occurrence clearly highlighted the specific effects of nutraceuticals as useful adjuvant in the treatment of cKSC-affected dogs, particularly for those animals poorly responsive or unresponsive to standard pharmacological therapy.

## Conclusions

To the best of our knowledge, this clinical evaluation represents first study that proposed the use of a specific antioxidant/anti-inflammatory ND as an optimal combination of ingredients with synergistic effects able to potentially exert an immune-modulating activity in combination with standard pharmacological treatments in cKCS.

The nutraceutical approach appears to significantly increase the eye’s tear production and to clinically ameliorate the conjunctival inflammation status as well as the corneal keratinization, corneal pigment density and mucus discharge in chronic cKCS dogs poorly responsive or unresponsive to immune-suppressive therapy.

The increased STT level in response to the proposed ND was in agreement with previously reported response to topical CsA and Tacrolimus [23, 98, 99]. Although we are unaware of the possible action mechanism of all ingredients, in particular for the phytotherapic extracts, we hypothesize that these substances and raw materials of the ND may exert a synergic action in the T-cell activation, possibly by preventing inflammatory gene transcription (IL-2, IL-3, IL-4, IFN- γ, TNF-α, GM-CSF, c-myc) [16, 100, 101].

Based on a possible mimicking action mechanism of all active substances with respect to CsA, we also hypothesized a reduced secretion of TNF-α by T cells. In this regard, TNF-α is known to increase mucin secretion from respiratory epithelial cells, thus it could possibly influencing the mucus production, corneal keratinization and conjunctival inflammation status [102, 103]. However, as observed by Hendrix et al. an overall significant improvement of clinical signs was not observed over time [93].

Intriguingly, our results seem to support the use of an anti-inflammatory/immune-modulating ND as an adjuvant to drug therapy in those cKCS dogs unresponsive to pharmacological treatment, in order to achieve analogue results of the responsive subjects (Moore et al., [94], Hendrix et al., [93]). Therefore, our investigation highlights the relevance of the possible administration of antioxidant/anti-inflammatory nutraceutical diet to cKCS dogs as useful adjuvant of immunosuppressive therapy.

The combination of a pharmacological treatment with a specific diet (Ocu-GLO Rx™) was also recently assessed by Williams et al. who successfully delayed the cataract formation in dogs with diabetes mellitus [104]. Specifically, the diet consisted in a mixture of a aldose reductase inhibitor, a glutathione regenerator alpha lipoic acid, grape seed extract, carotenoids, omega-3-fatty acids, and coenzyme Q10 which was provided to diabetic dogs as far as these developed lens opacification. Mean time without change in lens opacification was 278 ± 184 days with Ocu-GLO Rx™ and 77 ± 40 days in the placebo group.

In our treatement approach, the combination of several nutraceuticals, such as fish hydrolised proteins, rice carbohydrates, *Cucumis melo*, *Ascophyllum nodosum*, Astaxanthin, *Aloe vera*, *Carica papaya*, *Punica granatum*, *Camellia sinensis*, *Polygonum L*., *Curcuma longa*, *Piper nigrum*, zinc and a omega3/6 polyunsaturated fatty acids (1:0.8 ratio), appears to exert beneficial immune-modulating effects on the clinical status of cKCS dogs. These data seams to confirm the action of nutraceutical diet on immune system modulation reducing Th1 and inproving TReg [35].

These plants and substances, widely used in traditional medicine, have been already shown to exert some intriguing antioxidant and anti-inflammatory activities in ocular tissues. In this regard, it is worth noting that *Camellia sinensis* extract was effective in conjunctival inflammation treatment [105] and *Curcuma longa* in several ocular diseases (chronic anterior uveitis, diabetic retinopathy, glaucoma, age-related macular degeneration and dry eye syndrome) [106, 107]. In addition, zinc was observed to reduce the progression of the age-related macular degeneration by the inhibition of the complement activation on retinal pigment epithelium cells [108] and omega 3 -6 fatty acids were closely correlated to development of vision and protection of eyes [109, 110].

The antioxidant/anti-inflammatory effects likely possessed by the mixture based on all these nutraceuticals in the diet supplementation seems to specifically reduce the immune-mediated ocular symptoms, particularly in those cKCS dogs that were poor responsive or unresponsive to classical immune-suppressive drugs.

In this regard, the pharmacological treatment alone was able to increase lachrymal production, while the increment was strongly higher and persistent when drugs were combined with the ND. Likewise, conjunctival inflammation was significantly reduced more in dogs belonging to ND group (receiving drugs in combination with nutraceutical supplemented diet) than in the SD group (receiving only the medical treatments). In addition, it is of relevance that corneal pigment density and mucus discharge were improved only in dogs belonging to the ND group. Finally, the occurrence of symptom relapsing, upon the suspension of nutraceutical diet, and of clinical amelioration, after its reintroduction, fosters the hypothesis of a possible therapeutical benefit of this nutraceutical diet in animal as well as in human ocular diseases [111, 112]

Taken in all, our results suggest that association of classical drug therapy with a nutraceutical diet with potential antioxidant/antiinflammatory and immune-modulating activities induce a significant amelioration of clinical signs and symptoms in keratoconjunctivitis sicca. Moreover, all symptoms appeared dependent on immune-mediated mechanisms. In this regard, the lachrymation impairment can be altered by an inflammatory condition of lachrymal gland and related ducts.

Therefore, it is reasonable to hypothesize that metabolic changes could affect immune response orchestration in a model of immune-mediated ocular disease, as represented by keratoconjunctivitis sicca, in dogs and, in a translational perspective, by Sjögren’s syndrome in humans.

### Study limitations

This research has some study limitations. For instance, neither the inflammatory cytokines present in the serum of dogs affected by KCS nor the percentage of regulatory T cells in the blood were evaluated. Ongoing experiments are characterizing the inflammatory cytokine release as well as the presence of Treg cells in peripheral blood. Moreover, preliminary results have evidenced that it is really hard to find in blood those alterations likely present in a well-defined peripheral tissue and body district, as represented by the eye.
