# Erratum to: Clinical evaluation of a nutraceutical diet as an adjuvant to pharmacological treatment in dogs affected by Keratoconjunctivitis sicca

**DOI:** 10.1186/s12917-016-0848-8

**Published:** 2016-12-05

**Authors:** Simona Destefanis, Daniela Giretto, Maria Cristina Muscolo, Alessandro Di Cerbo, Gianandrea Guidetti, Sergio Canello, Angela Giovazzino, Sara Centenaro, Giuseppe Terrazzano

**Affiliations:** 1 Clinica Veterinaria Porta Venezia, via Lambro 12, 20121 Milan, Italy; 2 Clinica Veterinaria Cartesio, viale Olanda 3B, Melzo, 20066 Milan, Italy; 3 Ambulatorio Veterinario Canonica, via Canonica 36, 20154 Milan, Italy; 4 School of Specialization in Clinical Biochemistry, “G. d’Annunzio” University, Chieti, Italy; 5 Research and Development Department, SANYpet S.p.a., Bagnoli di Sopra, Padua, Italy; 6 Department of Science, University of Basilicata, Via Sauro, 85, 85100 Potenza, Italy; 7 Department of Translational Medical Sciences, University of Naples Federico II, Via Pansini, 5, 80131 Naples, Italy

## Erratum

*n.b. The error described below was mistakenly carried forward by the production team handling this article, and thus was* ***not*** *the fault of the authors.*

The original version of this article [1] contained errors in Fig. 2 whereby Fig. 2b, c, d and e were missing the asterisks describing the P value relating to the difference in score between the ‘Control’ and ‘Treatment’ groups at the ‘Post-treatment’ stage.

**Fig. 2:**
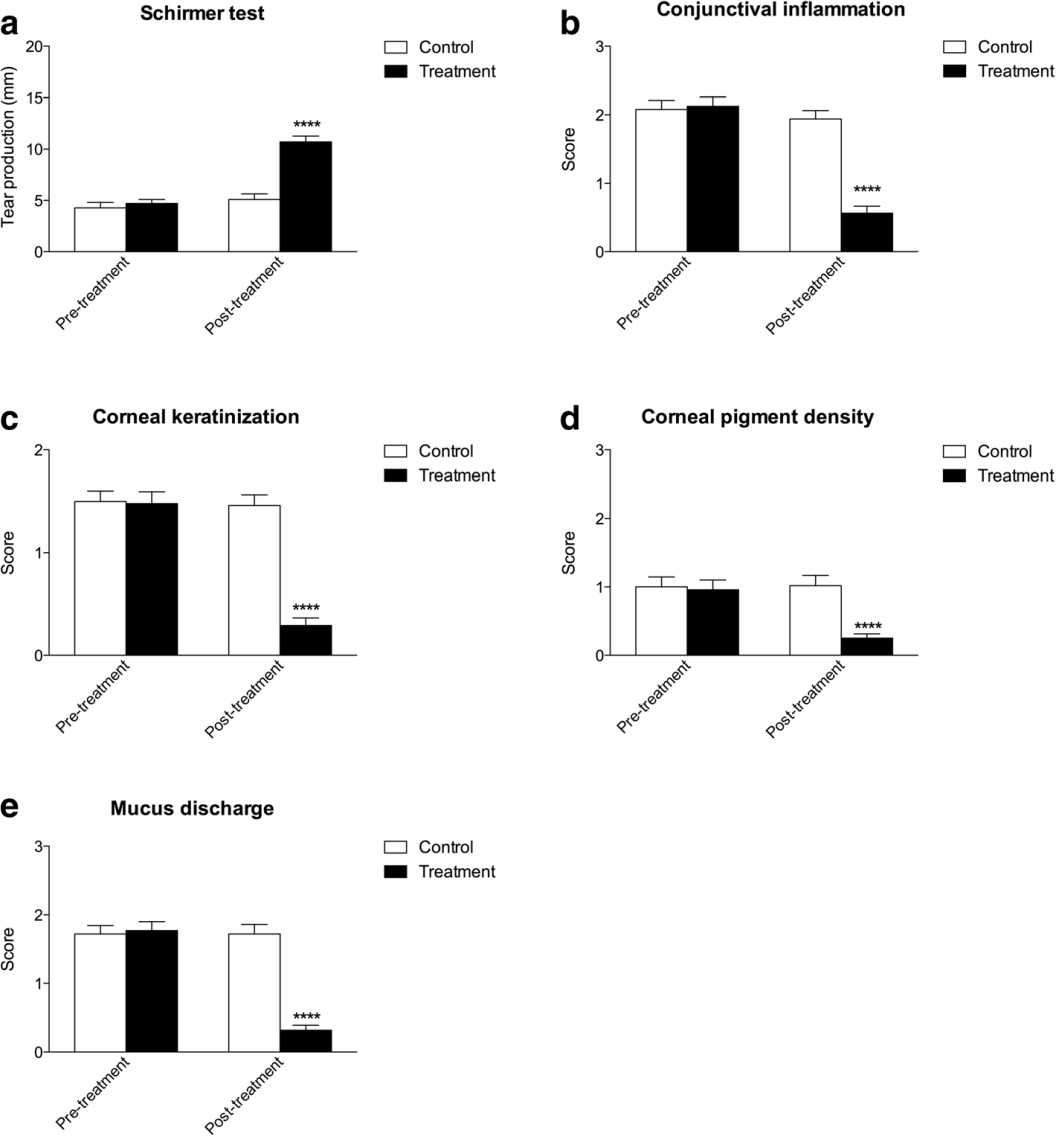
**a** mean tear production (STT) in mm/min before and after 60 days treatment for ND and SD group, STT values resulted significantly increased (****P < 0.0001) in ND group at the end of treatment, **b** Mean conjunctival inflammation scores before and after 60 days treatment for ND and SD group, a significant decrease (****P < 0.0001) was observed in ND group at the end of the treatment; **c** mean corneal keratinization scores before and after 60 days treatment for ND and SD group, a significant decrease (****P < 0.0001) was observed in ND group at the end of the treatment; **d** mean corneal pigment density scores before and after 60 days treatment for ND and SD group, a significant decrease (****P < 0.0001) was observed in ND group at the end of the treatment; **e** mean mucus discharge scores before and after 60 days treatment for ND and SD group, a significant decrease (****P < 0.0001) was observed in ND group at the end of the treatment

The article has now been updated to incorporate the correct version of Fig. 2, which now displays these asterisks for each graph where appropriate.

The updated Fig. 2 can be seen below for reference.
